# Effects of Aqueous Isotopic Substitution on the Adsorption Dynamics and Dilational Rheology of β-Lactoglobulin Layers at the Water/Air Interface

**DOI:** 10.1021/acs.jpcb.3c08417

**Published:** 2024-03-12

**Authors:** Georgi G. Gochev, Emanuel Schneck, Reinhard Miller

**Affiliations:** † Jerzy Haber Institute of Catalysis and Surface Chemistry, Polish Academy of Sciences, 30239 Krakow, Poland; ‡ Institute of Physical Chemistry, Bulgarian Academy of Sciences, 1113 Sofia, Bulgaria; § TU Darmstadt, Institute for Condensed Matter Physics, 64289 Darmstadt, Germany

## Abstract

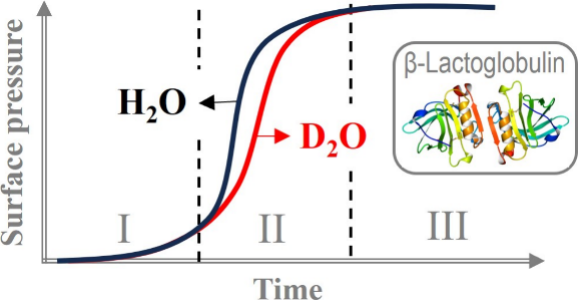

The effect of the degree of isotopic substitution of the aqueous medium on the adsorption kinetics and the surface dilational rheological behavior at the water/air interface of the globular protein β-lactoglobulin was investigated. Aqueous solutions with fixed concentrations of 1 μM protein and 10 mM hydrogenous buffer with controlled pH 7 were prepared in H_2_O, D_2_O, and an isotopic mixture of 8.1% v/v D_2_O in H_2_O (called air contrast matched water, ACMW). Using a bubble shape analysis tensiometer, we obtained various experimental dependencies of the dilational viscoelasticity modulus E as a function of the dynamic surface pressure and of the frequency and amplitude of bubble surface area oscillations, either in the course of adsorption or after having reached a steady state. In general, the results revealed virtually no effect from substituting H_2_O by ACMW but distinct albeit relatively weak effects for intermediate adsorption times for D_2_O as the aqueous phase. In the final stage of adsorption, established after around 10 h, the equilibrium adsorption and the dilational rheological behavior of all protein layers under investigation are only very weakly affected by the presence of D_2_O. The obtained results help to design experimental protocols for protein adsorption studies, for example, by neutron reflectivity.

## 1 Introduction

The use of heavy water (D_2_O) as an aqueous medium is a prerequisite for advanced protocols in various experimental characterization methods, for example, neutron scattering and some spectroscopic techniques such as nuclear magnetic resonance and infrared spectroscopy.^1−6^ The use of D_2_O instead of H_2_O in aqueous protein bulk studies was very recently discussed in a perspective article by Giubertoni et al.^6^ The basis of the differences between the solvation effects of H_2_O and D_2_O is that the hydrogen bond (OH**···**O) is slightly weaker than the “hydrogen bond” in D_2_O (OD**···**O). Such a solvation effect may influence to a certain degree the stability/flexibility of protein globules,^6−9^ which, in turn, may lead to changes in some physicochemical properties of globular proteins either in the bulk or when adsorbed at interfaces, for instance, the denaturation and aggregation kinetics in the bulk^10−13^ as well as the adsorption kinetics at interfaces.^14,15^ The observed inhibitory effect of D_2_O on the activity of some enzymes is worth noting.^11^

The present work is related to the role of proteins in colloid stability and protein adsorption at interfaces, in particular. Grunwald et al.^14^ used surface plasmon resonance (SPR) to measure the adsorption kinetics at a solid hydrophobic surface of four proteins adsorbed from buffer solutions in H_2_O or D_2_O. The authors found a distinct isotopic effect: generally, the protein adsorption kinetics are slower in D_2_O, but to a different degree for the different proteins. Ganzevles et al.^15^ reported that the adsorption of β-lactoglobulin (BLG) at the water/air (W/A) interface is up to 3 times slower in D_2_O than in H_2_O. However, both studies reveal that the observed adsorption kinetics differences manifest only in the initial stage of adsorption and at longer times the isotopic effect fades away.

In order to obtain deeper insights into the effects of the isotopic substitution in the aqueous medium on protein adsorption as well as in relation to our previous neutron reflectivity work on the adsorption dynamics and structure of BLG layers at the W/A interface,^2^ we performed dedicated surface tension and surface dilational rheometry measurements for buffered BLG solutions in H_2_O, D_2_O, and air contrast matched water (ACMW).

## 2 Experimental Section

In all experiments, a powder sample of native BLG (M_w_ ≈ 18.3 kg/mol), kindly supplied by U. Kulozik (TU Munich, Germany) was used.^16^ The sample is ∼93.5% dry matter, which contains ∼98.9% total protein (from which the BLG content is >99%, BLG-A/BLG-B ≈ 1.22), ∼0.7% salts, and traces of lactose (<0.05%). Citric acid (ACS reagent, ≥99.5%, 251275 Merck) and Na_2_HPO_4_ (≥99.99% trace metals basis, 731478 Merck) were used to prepare BLG solutions at 10 mM buffer and at pH 7.0 in three types of aqueous media with different degrees of H–D isotopic substitution (hereafter called “isotopic contrasts”): (1) Milli-Q H_2_O, (2) D_2_O (Merck), and (3) 8.1% v/v D_2_O in H_2_O (ACMW). The pH value of 7.0 was adjusted in either medium by measurements with a commercial glass-electrode-based instrument (standardized to read pH for H_2_O solutions) without correction for pD (pD ≈ pH + 0.4).^17^ According to previous studies, we do not expect appreciable effects of changes in the pH range of 7.0–7.4 on the adsorption and dilational rheology behavior of BLG at the concentration studied (1 μM).^18,19^ Stock solutions of BLG with a concentration of C = 100 μM were prepared in each aqueous medium; to eliminate low-molecular-weight contamination, the protein stock solutions were purified with activated charcoal (BLG/charcoal mass ratio 1/3, stirred for 20 min)^20^ and filtered through a 0.45 μm pore size protein-nonbinding filter. These stock solutions were left to rest overnight in a refrigerator in order to reach full hydration as well as the entire exchange of labile hydrogens in the protein molecules. All measurements in this work were performed with diluted aliquots of C = 1 μM (∼1.83 × 10^–3^ wt %; ∼1.83 × 10^–2^ mg/mL) in the respective aqueous isotopic contrast.

Surface tension γ was measured with a drop/bubble profile analysis tensiometer PAT-1 M (Sinterface Technologies, Germany). In a typical experiment, a buoyant air bubble is formed at the tip of a hook-shaped steel capillary immersed in solution. In the present experiments, the bubble’s area A_0_ = 25 mm^2^ was kept constant in the course of adsorption. The accuracy of the tensiometry measurements γ(t)_A_0__ was always better than ±0.2 mN/m in all experiments. In the experimental results, the surface tension and the corresponding surface pressure Π are used interchangeably, Π = γ_0_ – γ, where γ is the measured value for a BLG solution and γ_0_ is the value for the protein-free buffers. The latter was found to vary between 72.3 and 72.6 mN/m^2^ at room temperature (22–23 °C), and no appreciable effects of the different aqueous isotopic contrasts (H_2_O, ACMW, or D_2_O) were detected. For each isotopic contrast, at least two independent measurements were performed with a duration of 1–2 h each, and one or two longer measurements were performed for 20–22 h, where the surface pressure has reached a steady state (near-equilibrium conditions). Harmonic area oscillations were periodically applied in the course of adsorption, and the surface tension response γ(A(t)) was recorded (Figure S1 in Supporting Information). The absolute values for the frequency-dependent complex dilational viscoelasticity modulus
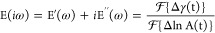
1and for the viscous phase angle ϕ^21−23^ were calculated by automatic calculation protocols integrated into the instrument’s software. In eq 1,  stands for the first harmonic Fourier transform and ω [rad·s] = 2πf [Hz] is the angular frequency associated with the oscillation frequency f in Hz. Parameters E′ and E′′ are the real (elastic) and imaginary (viscous) parts of the complex modulus E, for which the following relations apply:^21^
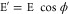
2a
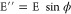
2bWe obtained sets of experimental data for different dependencies of the dilational rheology parameters  ≡ {E, ϕ, E′, E′′}

(t)_g,f_
(Π(t))_g,f_
(g)_g,Π_ {f sweeps}
(g)_f,Π_ {g sweeps}

where g ≡ ΔA/A_0_[× 100%] is the amplitude of area deformation. Further information about the experimental details and the obtained results is given in the following section and in the Supporting Information.

## 3 Results

The dynamic dependencies (t)_g,f_ were obtained by periodical applications during the adsorption process of a set of two single oscillations at a constant frequency of f = 0.1 Hz and at two amplitudes: g_1_ = 2.5 ± 0.5% and g_2_ = 6.5 ± 0.5%. An overall standard deviation of ±0.5% was calculated from all of the measurements made in this study. Combining these data with the corresponding data for dynamic surface pressure Π(t) allowed the dynamic rheological dependencies (Π(t))_g,f_ to be constructed. The experimental oscillation protocol is illustrated in Figure S2 (Supporting Information). The sets of dependencies (f)_g,Π_ and (g)_f,Π_ were obtained by applying more complicated oscillation protocols either during the adsorption process or at a steady state reached after 20–22 h; these protocols are explained in detail in the Supporting Information (Figure S3). To accept the adequate application of any of the oscillation protocols, we strictly kept the following necessary condition: the values of the surface pressures Π_A_0__ measured at undisturbed area A_0_ in the beginning and at the end of a given oscillation protocol should not differ by more than 0.5 mN/m.

Within the used frequency range (0.01–0.1 Hz), the viscoelastic behavior of BLG at W/A interfaces is predominantly elastic and the real part E′ can be approximated by the modulus E.^19,24^ In a previous study, we found that E*′*(Π)_g,f_ ≈ E(Π)_g,f_ ≈ E_0_(Π) (E_0_ is the high-frequency limiting or Gibbs elasticity), which stands for the linear viscoelasticity regime of a steady-state BLG monomolecular adsorption layer (up to Π ≈ 20 mN/m).^25^ All of the studied BLG adsorption layers in the present study fulfill these conditions; therefore, for the sake of simplicity, in the following text we present results only for the dilational modulus E; the data for E′ are omitted, and the corresponding data for ϕ and E′′ are shown in the Supporting Information.

### 3.1 Experimental Approach and Types of Results

**Figure 1:**
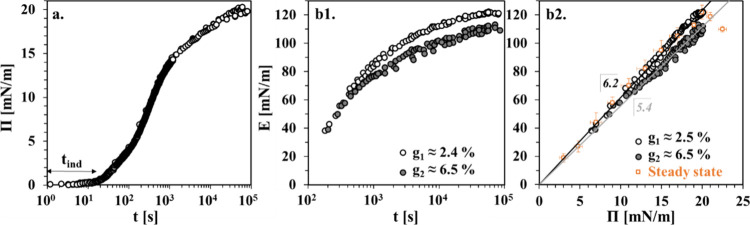
Results for BLG in H_2_O from two short (1–2 h) and two long (20–22 h) independent measurements. (a) Dynamic surface pressure Π(t); t_ind_ is the induction time. (b1) Dynamic dependencies of the dilational viscoelasticity modulus E(t)_g,f_ at f = 0.1 Hz and at oscillation amplitudes of g_1_ and g_2_ and (b2) the corresponding E(Π(t))_g,f_ dependencies; the straight lines through the symbols are linear regressions (details are given in the text). For comparison purposes, included are E(Π)_g,f_ data from the literature measured at a steady state (linear viscoelasticity regime, f = 0.1 Hz).^25^

**Figure 2:**
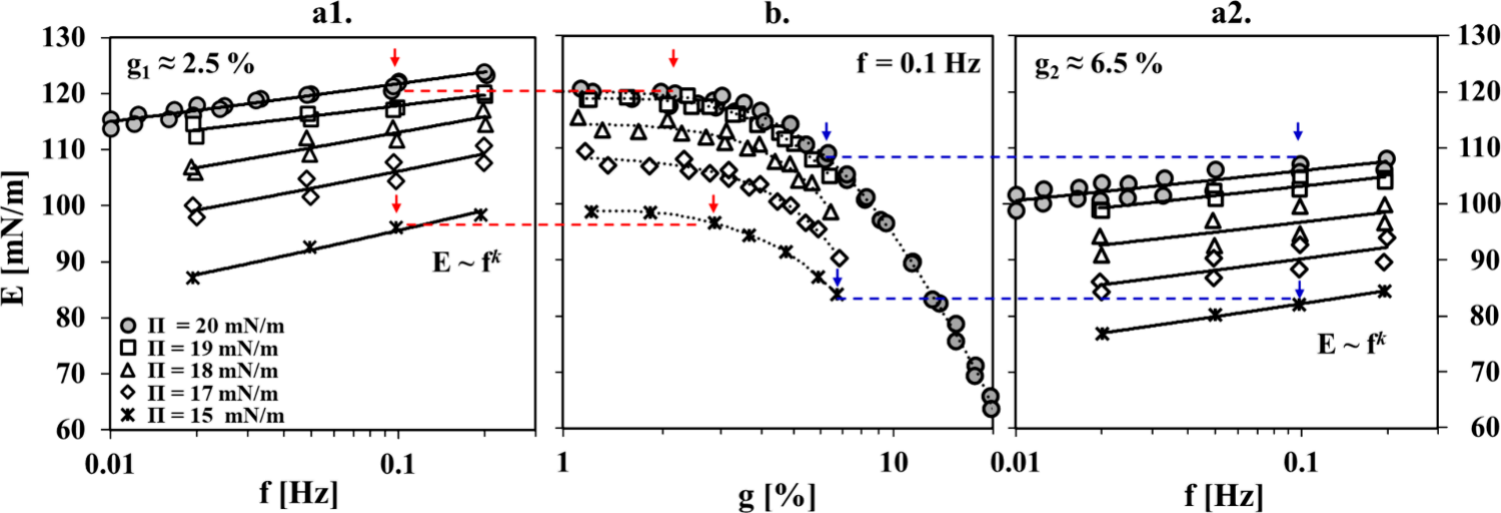
Results for the complex dilational viscoelasticity modulus Ε obtained from the same experiments as those in Figure 1. (a1, a2) Frequency dependencies of Ε(f)_g,Π_ at different surface pressures Π and at area deformation amplitudes g_1_ and g_2_. (b) Area deformation amplitude dependencies E(g)_f,Π_ at different surface pressures Π and at a frequency of f = 0.1. The solid lines through the symbols in (a1, a2) follow power law E ≈ f^*k*^. The dotted lines in (b) are guides to the eye, and the straight horizontal dashed lines connect data points E_f,g_ (indicated with arrows) measured independently under identical area oscillation conditions.

**Figure 3:**
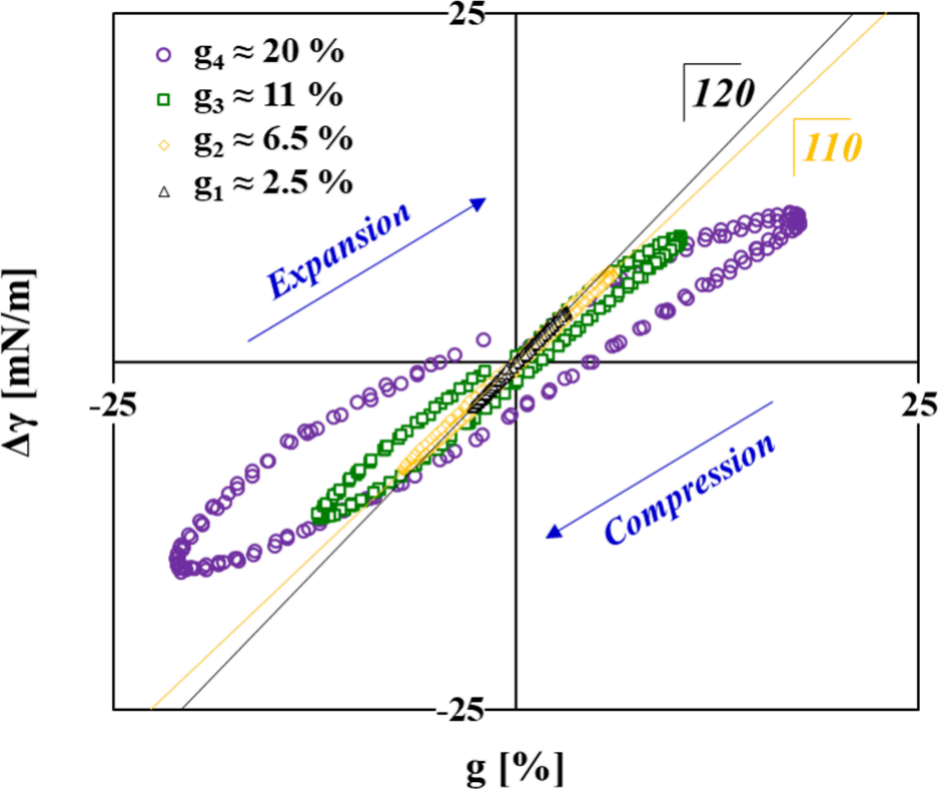
Results at steady state (Π ≈ 20 mN/m) from the two long (20–22 h) experiments in Figure 1. Lissajous plots Δγ–g constructed from the raw data γ(A(t)) measured during bubble oscillations in large-range g sweeps. The straight lines are linear regressions to the data for g_1_ and g_2_ with slopes of 120 and 110, respectively.

To illustrate the experimental details and the different types of obtained data, we present in Figures 1–3 the results for BLG in H_2_O solutions.

Figure 1 presents experimental data for the dynamic surface pressure and dynamic dilational viscoelasticity modulus. The data for the other rheological parameters ϕ and E′′ are given in Figure S4 (Supporting Information). The Π(t) curves from the different measurements overlap well on a master curve (Figure 1a), which displays the typical sigmoidal shape observed for protein solutions, where the so-called induction time t_ind_ is defined by the onset of measurable surface pressure^18,26,27^ (in the present case, t_ind_ ≈ 10 s). The E data shown in Figures 1b1 and b2 reveal that for values of E < 60 mN/m, corresponding to Π < 10 mN/m, the modulus E is virtually insensitive to changes between the two applied oscillation amplitudes. Similar behavior was also observed for other globular proteins (ovalbumin and bovine serum albumin (BSA)) in dilational rheometry experiments for amplitudes g of up to 15%.^28^ At E > 60 mN/m, the effect of g becomes significant. The E(Π(t))_g,f_ dependencies in Figure 1b2 refer to a linear regression.^18,28,29^ The slope dE/dΠ = 6.2 for the data at g_1_ ≈ 2.5% (linear viscoelasticity regime) corresponds exactly to the run of the E_0_(Π) dependence as calculated in ref (25) by fitting a theoretical model to experimental data E(Π)_g,f_ for steady-state BLG adsorption layers (included in Figure 1b2 for comparison). The E_0_(Π) dependence is actually a surface equation of state (EoS) derived from the classical thermodynamic expression Π(Γ), where Γ is the surface excess.^25^ Apparently, the adsorption kinetics and the linear viscoelasticity behavior of the dynamic BLG adsorption layers investigated here are governed by the steady-state (near-equilibrium) EoS. For g_2_ ≈ 6.5%, a slightly lower slope of dE/dΠ = 5.4 was found. Such a dependence of the dilational viscoelasticity modulus on the oscillation amplitude has previously been explained by the nonlinearity of the EoS.^30^

The data for ϕ and E′′ (Figure S4 in Supporting Information) show a maximum at Π ≈ 15 mN/m, which was interpreted earlier as a result of weak relaxation processes at the interface taking place during a transient step in the course of adsorption.^19^ The observed rheological behavior is conveniently illustrated by presenting the viscous term E′′(Π(t))_g,f_ as a function of the modulus E(Π(t))_g,f_, and such plots are shown in Figure S5 (Supporting Information).

Figure 2 presents the experimental dependencies Ε(f)_g,Π_ and E(g)_f,Π_, and the corresponding results for ϕ and E′′ are shown in Figure S6 (Supporting Information). In the present work we employed a new experimental approach and measured these dependencies in the course of the adsorption process by applying appropriate oscillation protocols (i.e., narrow f sweeps and g sweeps for surface pressures in the range of 15–19 mN/m), which was chosen for the following reasons: the surface pressure of Π ≈ 15 mN/m is reached after about half an hour of adsorption, and at this stage, the rate of increase of Π is already sufficiently slow to allow for adequate application of narrow f sweeps and g sweeps (Figure S3a in Supporting Information). The wider f-sweep and g-sweep protocols (Figures S3b,c in Supporting Information) were applied once the adsorption layer had reached a steady state (Π ≈ 20 mN/m). This approach provided reliable data as illustrated in Figure 2, where E_f,g_ values measured independently under identical area oscillation conditions (f, g) are in excellent agreement. In general, the shape of each of these two types of dependencies seems to be weakly affected by an increase in the surface pressure in the monitored Π range.

Figure 2b reveals a transition from a linear to a nonlinear viscoelasticity regime at a transition amplitude of g_tr_ ≈ 3%, which agrees well with previous results, where g_tr_ ≈ 4% was reported for BLG adsorption layers either at W/A interfaces^31^ or at water/MCT oil interfaces.^24^ Furthermore, apparently g_tr_ is virtually independent of pH (3–7) and Π (15–20 mN/m) [Supporting Information to ref (25)]. Hence, the amplitudes g_1_ ≈ 2.5% < g_tr_ and g_2_ ≈ 6.5% > g_tr_ fixed in other types of dependencies belong to the linear and nonlinear viscoelasticity regimes, respectively. For g > g_tr_, increasing g leads to a linear decrease (R^2^ ≈ 0.99, shown in Figure S8 in the Supporting Information) of the dilational viscoelasticity modulus E.^25,31^ This run corresponds to gradual increases of ϕ and E′′ (Figure S6 in the Supporting Information).

However, it may appear that the standard output modulus (hereafter denoted E_FT_) from bubble shape analysis tensiometers, based on the first harmonic Fourier transform analysis of the surface tension response to area oscillations,^21^ could be insufficient to describe the rheological behavior of highly nonlinear viscoelastic interfacial systems, for instance, due to in-plane deviatoric stresses among other factors present in the surface tension response.^32^ Any information eventually encoded in higher harmonics remains inaccessible, and extracting such information requires the application of methods other than the first harmonic Fourier transform analysis.^33^ Therefore, we furthered the exploitation of the raw γ(A(t)) oscillation data by employing the Ewoldt formalism^34^ as developed for the case of surface (shear and dilational) rheology by Sagis and co-workers.^32,35,36^ In this approach, the stress response E̅ to a surface expansion/compression strain is decomposed into two large-strain moduli E̅_LE_ and E̅_LC_ and two minimum-strain moduli E̅_ME_ and E̅_MC_, where E̅_LE_ and E̅_ME_ correspond to area expansion and E̅_LC_ and E̅_MC_ correspond to area compression. (For further details, see the Supporting Information and refs (32−36)). Then, two strain-stiffening factors are defined:
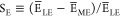
3a
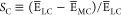
3bThese S factors are conveniently used as measures for the degree of elastic intracycle nonlinearity both in expansion S_E_ and in compression S_C_ as (1) S = 0 for linear viscoelasticity behavior, (2) S > 0 for strain-hardening, and (3) S < 0 for strain-softening.

The use of this approach is based on the analysis of so-called Lissajous plots.^24,32−36^ We constructed experimental Lissajous plots for four exemplary amplitudes (g_1_–g_4_), and they are presented in Figure 3 in terms of Δγ vs g, where Δγ = γ_t,A_ – γ_A_0__ (γ_t,A_ is the instantaneous surface tension at time t and over the corresponding area A and γ_A_0__ is the reference quasi-static surface tension over the undisturbed area A_0_).

The analysis of the data in Figure 3 is illustrated in detail in Figure S7 (Supporting Information); the numerical analysis was made in Microsoft Excel within an accuracy of ±1 mN/m. For g_1_ ≈ 2.5% < g_tr_, the data collapse well on a master curve E̅ ≡ E̅_LE_ = E̅_ME_ = E̅_LC_ = E̅_MC_ = 120 mN/m. Such a shape of the Lissajous plot indicates the highly elastic linear rheological behavior of the adsorption layer. As expected, the obtained value is practically the same as the one obtained from the Fourier transform analysis E_FT_ = 120 mN/m. For g_1–3_ > g_tr_, the shapes of the Lissajous plots suggest a nonlinear asymmetric stress response. However, for g_2_ ≈ 6.5%, this is still quite weakly pronounced, and it was possible to consider a data treatment in the same way as for g_1_; the obtained value for the E̅ modulus of 110 mN/m (Figure 3) is very close to E_FT_ = 107 mN/m. However, a more detailed analysis yielded values for the four decomposed terms of E̅. For the higher amplitudes g_3_ ≈ 11% and g_4_ ≈ 20% (the highest one used in this work), the nonlinear asymmetric stress response is more pronounced as evidenced by their shapes in Figure 3. Note that Δγ widening of the loop shape of the Lissajous plots for increasing strains g corresponds to an increase in the viscous contribution E′′ to the complex dilational viscoelasticity modulus (Figure S6b in the Supporting Information). The obtained results for the moduli E̅_LE_, E̅_ME_, E̅_LC_, and E̅_MC_ are summarized in Figure S8 (Supporting Information) in terms of linear regression over the data sets g_1_–g_4_ for each type of modulus (tabulated values are in Table S1 in the Supporting Information).

For the case of H_2_O solutions, the results revealed that the values for the two S factors are very close, namely S_E_ ≈ S_C_ ≈ −0.05 ± 0.01. The negative sign of these values means that the examined BLG adsorption layers exhibit strain-softening rheological behavior, but such a low absolute value suggests that the degree of elastic nonlinearity remains comparatively low up to the highest amplitude of 20% used in our study. Similar behavior was also found for other proteins at W/A interfaces and for even larger amplitudes of up to 60%,^36^ while for example, for oligofructose esters, the absolute values of the S factors are higher by an order of magnitude and moreover have different signs (S_C_ > 0 and S_E_ < 0), which points to more complex nonlinearity behavior.^35^ Such behavior observed for different surface-active species is attributed to changes in the interfacial microstructure.^32,37^ Hence, the low degree of dilational stress response nonlinearity of protein monolayers should be the result of a relatively homogeneous 2D structure, which was indeed revealed by neutron reflectivity experiments with BLG in D_2_O.^2,38^

We now discuss the E(f)_g,Π_ dependences in Figure 2a1,a2. In general, the frequency dependence of the dilational viscoelasticity modulus of protein layers is relatively weak in the studied f range (0.01–0.1 Hz)^18,28,39,40^ as compared, for example, to protein aggregates or low-molecular-weight surfactants.^35,41^ Concerning BLG, note that in refs (18) and (39) the experiments were performed in the nonlinear viscoelasticity regime, while in ref (40) the authors have not indicated the oscillation amplitude. The reason for such a weak effect of the frequency on the dilational viscoelasticity modulus of BLG adsorption layers at W/A interfaces should be that the considered f range is in the vicinity of the high-frequency limit E_0_.^25,39^

The Ε(f)_g,Π_ data can be empirically described by a simple power law E ∝ f^*k*^. For casein micelles the exponential factor is *k* ≈ 0.35, while for low-molecular-weight surfactants it might be even higher (*k* ≈ 0.5), both values suggesting a diffusion-controlled exchange of matter mechanism according to the Lucassen and van den Tempel formalism.^35,41^ Values of *k* ≈ 0.1 were reported for adsorption layers of wheat or lentil protein extracts at Π ≈ 20–21 mN/m.^42^ In the present results, the factor *k* is somewhat lower. Furthermore, increasing the surface pressure from 15 to 20 mN/m leads to a slight decrease of the *k* values within the range 0.05–0.02 in approximately a linear manner (Figure S9 in Supporting Information). For all the cases of wheat and lentil protein extracts,^42^ and the here investigated BLG, such *k* values suggest a mechanism of interfacial processes during expansion/compression, which is different from diffusion-controlled exchange of matter. Indeed, comparison of a theoretical model for protein adsorption^43^ to experimental E(f)_g,Π_ data for equivalent BLG adsorption layers as in the present study revealed unrealistically low values of the used apparent diffusion coefficient (in the order of 10^–14^ m^2^/s) for surface pressures Π > Π*,^25^ where Π* is a critical value, which divides the surface pressure isotherm for proteins into a so-called precritical and postcritical range.

### 3.2 Comparison of the Results for H_2_O, ACMW, and D_2_O

**Figure 4:**
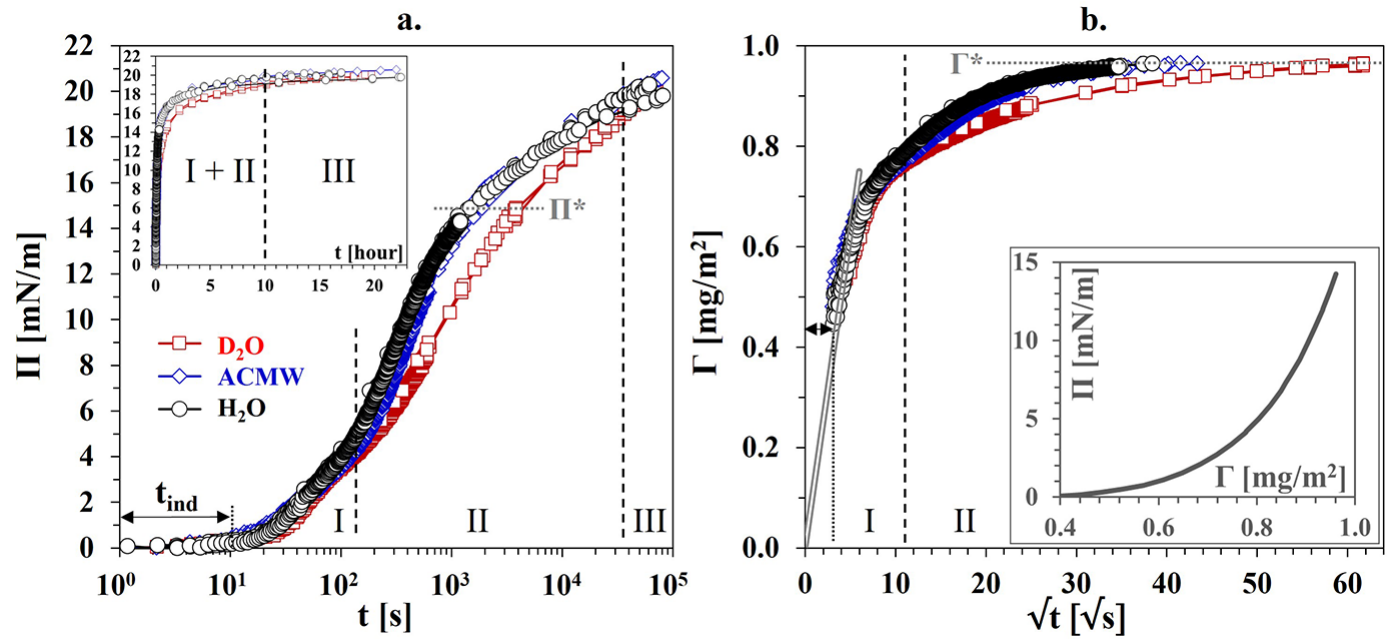
Adsorption kinetics of BLG solutions at C = 1 μM, C_buff_ = 10 mM, and pH 7 in H_2_O, ACMW, and D_2_O. The vertical dashed lines divide temporal regions I, II, and III (explained in the text); double-ended arrows indicate the induction time t_ind_. (a) Dynamic surface pressure Π(t) (inset: note the time scale in hours); lines through symbols are guides to the eye; Π* = 15.1 mN/m (details are given in the text). (b) Dynamic surface excess Γ(). The presented data correspond to the surface pressure range Π ≤ Π*, and the horizontal dotted line indicates the surface excess Γ* ≈ 0.97 mg/m^2^ at Π*. Lines through symbols are guides to the eye. Inset: surface equation of state Π(Γ) based on data from ref (25).

Figure 4a presents the results for the dynamic surface pressure Π(t) for the investigated BLG adsorption layers. The data are presented as three data sets corresponding to the three isotopic contrasts (H_2_O, ACMW, or D_2_O). Each data set is constructed from at least three independent measurements for different times of adsorption, including one or two long measurements (20–22 h). In Figure 4b, the dynamic surface excess is presented in terms of Γ(). These data are calculated from the measured Π(t) data through the surface equation of state Π(Γ) found in a previous study for the same H_2_O solvent conditions^25^ and shown in the inset of Figure 4b. Note that here we exclude any isotopic effects on the equation of state Π(Γ). The presented Γ() data are restricted to the surface pressure range Π ≤ Π*. This restriction is set here due to the fact that the theory for protein adsorption used in ref (25) is rigorously developed only for the precritical region of the surface pressure isotherm.^43^ For BLG under the same solvent conditions as in the present study, Π* = 15.1 mN/m was found.^25^

It is evident from the first glance at Figure 4a that the data for H_2_O and ACMW are very close, whereas the data for D_2_O distinctly deviate from them for adsorption times of between ∼2 min and ∼10 h. The whole experimental time window of adsorption can then be divided into three temporal regions as follows.

I (t: 0–2 min; Π ≈ 0–4 mN/m) Initial region including t_ind_. During the induction time, the protein molecules adsorb at the interface and form a “2D gaseous” phase. Note that the surface excess Γ increases during this stage due to adsorption.^44^ The origin of the onset of measurable surface pressures is a first-order 2D phase transition between “gaseous” and “liquid-expanded” states.^45^ For all measurements at different isotopic contrasts, t_ind_ ≈ 10 s. At t > t_ind_, the surface pressure rapidly increases as the rate of Π increase is the same for all data up to Π ≈ 4 mN/m (end of region I). According to a previous study,^25^ the dimensionless surface coverage θ of BLG at the end of the induction time is θ ≈ 41% (corresponding to the onset of the equation of state Π(Γ) at Γ ≈ 0.40 mg/m^2^ as shown in the inset of Figure 4b) and at the end of region I it is θ ≈ 77% (Γ ≈ 0.76 mg/m^2^). Figure 4b shows that the dynamic surface excess Γ() initially follows a linear run, which corresponds to a purely diffusion-controlled regime of adsorption. The simple relation  (sometimes called the “short time approximation”) is commonly used as an appropriate approximation for describing this initial stage of adsorption.^46−48^ This equation was fitted to the linear part of the Γ() plot with the diffusion coefficient D as the only fitting parameter, and a satisfactory fit was obtained with D = 5.2 × 10^–11^ m^2^/s. This value is comparable to literature data obtained by different methods for BLG solutions in H_2_O (D_H_2_O_ ≈ 8 × 10^–11^ m^2^/s^49,50^) or in D_2_O (D_D_2_O_ ≈ 9.4 × 10^–11^ m^2^/s^51^).
II (t: 2 min −10 h; Π ≈ 4–19 mN/m) Intermediate region characterized by a fast adsorption rate. Within this region, the isotopic effect is well pronounced for the case of D_2_O, where the rate of adsorption is decelerated as indicated by a bending of the Π(t) data curve (and the respective Γ() curve) toward lower surface pressures (lower surface excesses, respectively).
III (t: > 10 h; Π ≈ 19–20 mN/m) Final region characterized by a slow adsorption rate. At the end of region II, all of the dynamic surface pressure data for any isotopic contrast levels off at Π = 19.5 ± 0.4 mN/m; then in region III, the adsorption layers slowly relax as the surface pressure increases only slightly (with ∼1 mN/m for each data set) to steady-state values of Π = 20.2 ± 0.3 mN/m. The estimated standard deviations of these Π values include all measurements, and any discrimination of data for the different isotopic contrasts in region III cannot be clearly stated. Furthermore, the rheology data obtained within this final stage of the adsorption layers’ evolution are similar for a given isotopic contrast; therefore, in Figure 6 we present unified rheology data for each isotopic contrast as gathered within region III.

**Figure 5:**
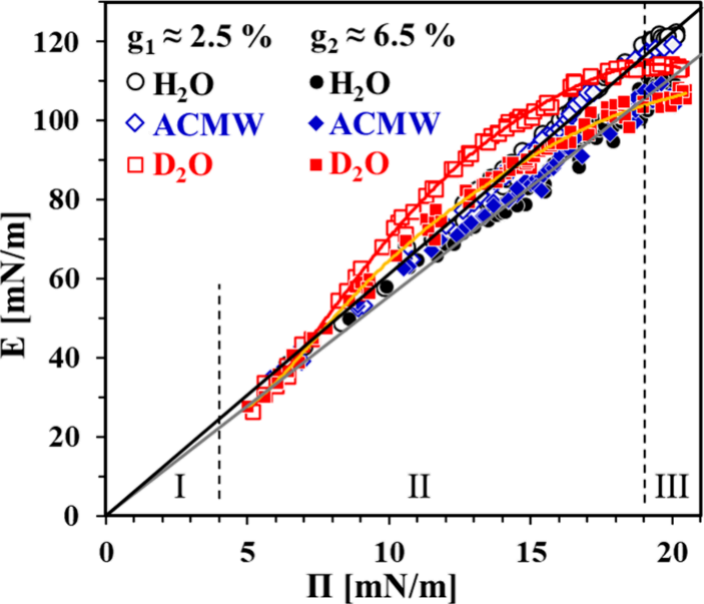
Dependencies of the dynamic dilational viscoelasticity modulus E(Π(t))_g,f_ at an oscillation frequency of f = 0.1 Hz and oscillation amplitudes of g_1_ ≈ 2.5% and g_2_ ≈ 6.5%. The straight lines are the same linear regressions as in Figure 1; the lines through the data for D_2_O are guides to the eye. Vertical dashed lines divide regions I, II, and III as defined in Figure 4.

Figure 5 presents E(Π(t))_g,f_ dependencies for H_2_O, ACMW, or D_2_O measured at two amplitudes g_1_ and g_2_. For each data set, the respective plots are constructed from experimental data from at least three independent measurements for different times of adsorption, including one or two long measurements (20–22 h). The relevant results for ϕ and E′′ and corresponding E′′(E)_g,f_ plots are shown in Figures S10 and S11 (Supporting Information).

**Figure 6:**
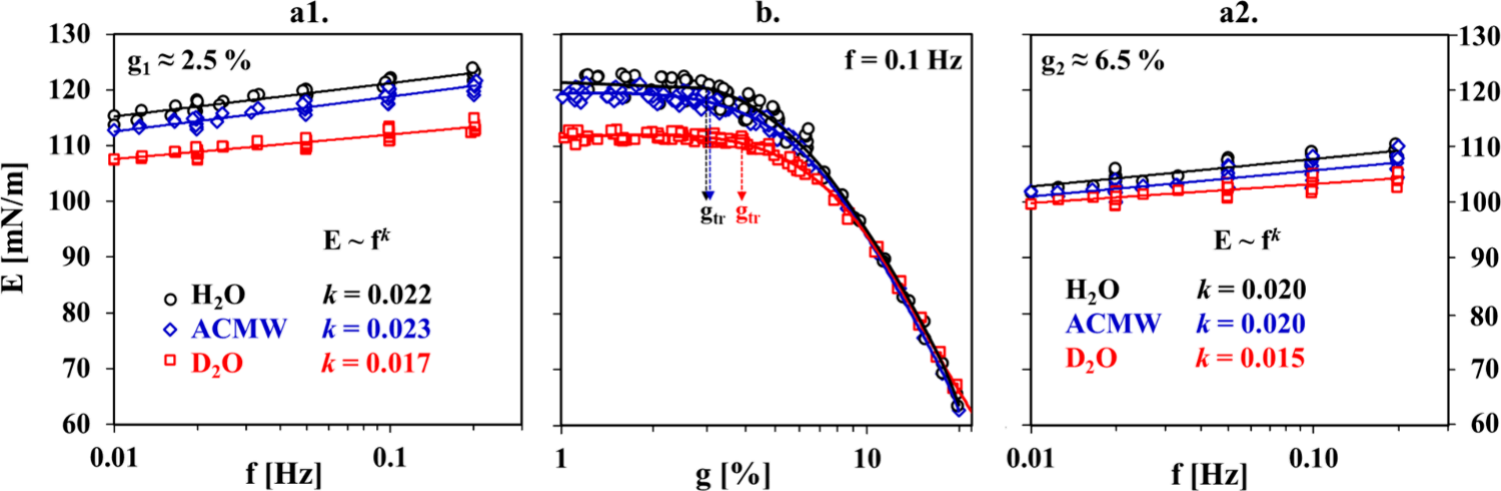
Dependencies of the dilational viscoelasticity modulus E on oscillation frequency f at g_1_ ≈ 2.5% (a1) and at g_2_ ≈ 6.5% (a2) and on oscillation amplitude g at f = 0.1 Hz (b). The data are gathered throughout region III (Π ≈ 19–20 mN/m). The lines through the symbols in (a1, a2) follow power law E ≈ f^*k*^. The lines in (b) are guides to the eye, and the arrows point to the transition amplitude g_tr_.

It is evident from the first glance at Figure 5 that for each case of the two used amplitudes the data for H_2_O and ACMW are virtually indistinguishable, whereas the respective data for D_2_O distinctly deviate from them in both regions II and III. On the other hand, the observed deviations of the data for D_2_O are much weaker for the higher-amplitude g_2_ than for the case of g_1_. The most striking point is the crossover of the D_2_O curves with the curves for H_2_O and ACMW (at g_1_). The appearance of such differences in the rheology data in region III is surprising, keeping in mind the similarity in the surface pressure results in this final temporal region (Figure 4). This issue is discussed in more detail in Section 4, considering also the E(f)_g,Π_ and E(g)_f,Π_ results (Figure 6).

Figure 6 presents E(f)_g,Π_ and E(g)_f,Π_ data recorded throughout region III (Π ≈ 19–20 mN/m); relevant results for ϕ and E′′ are shown in Figures S12 and S13 (Supporting Information). The data for region II (Π ≈ 15–19 mN/m) are not shown because they do not reveal any conceptual differences from the illustrative results for H_2_O in Figure 2 but do follow the shift in the E values for D_2_O outlined in Figure 5. It is worth noting that the exponential factors *k* in the power law fits of the frequency dependences E(f)_g,Π_ for ACMW and D_2_O vary with Π in the same manner as in the case of H_2_O explained above and illustrated in Figure S9 (Supporting Information).

The transition from a linear to a nonlinear viscoelasticity regime in the E(g)_f,Π_ data for ACMW occurs at virtually the same value of g_tr_ ≈ 3% as for H_2_O (Figure 6b). The difference in the average values  = 119 ± 1 mN/m and  = 120 ± 2 mN/m in the plateau regions of the linear viscoelasticity regime is within the scatter of the experimental data points; therefore, it can be neglected. On the contrary, the results for D_2_O reveal that the average plateau value of  = 112 ± 1 mN/m is distinctly lower (as evidenced also in Figure 5) and the transition amplitude (g_tr_ ≈ 4%) is apparently slightly higher than the common one for H_2_O and ACMW. However, for amplitudes g > 7% the E(g)_f,Π_ data for all isotopic contrasts overlap on a master curve with a scattering of only ±2 mN/m.

**Figure 7:**
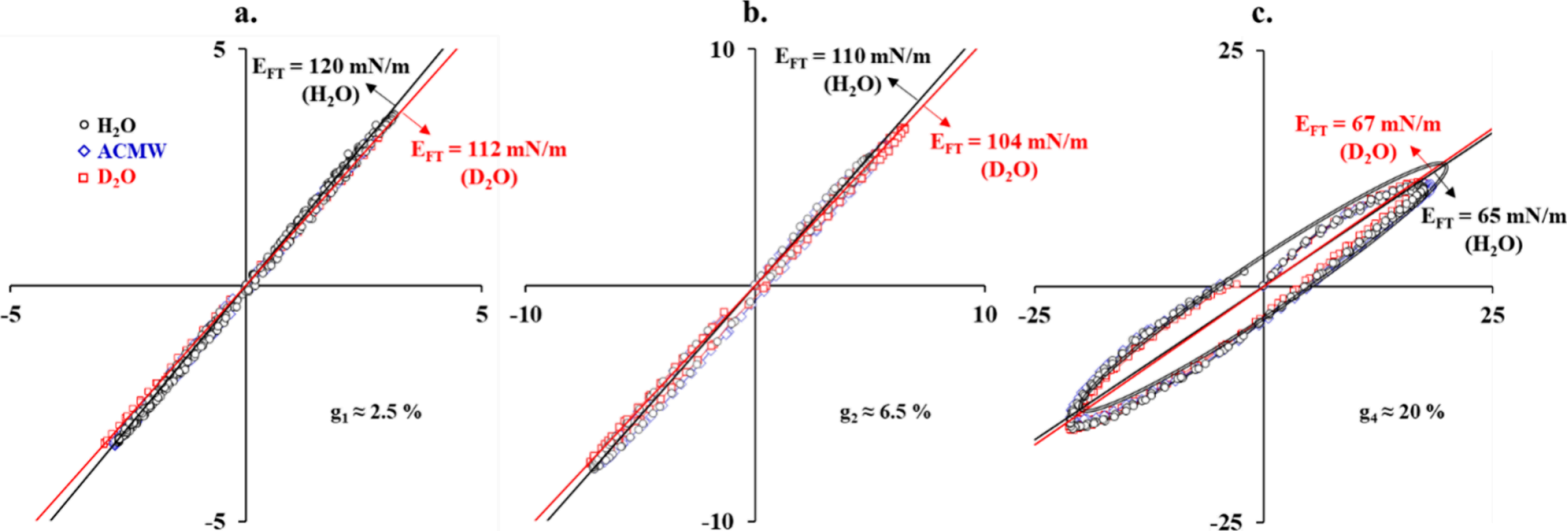
Lissajous plots Δγ–g (f = 0.1 Hz) at a steady state (Π ≈ 20 mN/m) constructed from the raw data γ(A(t)) measured during bubble oscillations in large-range g sweeps. The straight lines through the origin (0, 0) are the long axes of the elliptic contours, which are the first harmonic Fourier transform fits to the raw data (such an example is shown in c).

Figure 7 presents Lissajous plots Δγ–g at a steady state (Π ≈ 20 mN/m) for three (g_1_, g_2_, and g_4_) of the four exemplary amplitudes considered above (Figure 3). For comparison purposes, the results from the first harmonic Fourier transform analysis are illustrated as straight lines (which are the long axes of ellipses;^21^ an example for H_2_O is shown in Figure 7c). For the sake of simplicity, the analysis of the Lissajous plots is not presented graphically, but the obtained results for the E̅ moduli are tabulated in Table S1 (Supporting Information). The values obtained for the S factors reveal that in comparison to H_2_O and ACMW, the adsorption layers for D_2_O exhibit a slightly stronger strain-softening effect in compression (S_C_ ≈ −0.09) and lack strain-stiffening effects in expansion (S_E_ ≈ 0). However, these differences are relatively small, and any further interpretation would be rather speculative.

## 4 Discussion

In their SPR study on the adsorption kinetics of four proteins (streptavidin, glutathione-S-transferase, ribonuclease A, and BSA) at a solid hydrophobic surface, Grunwald et al.^14^ observed a general slow down in the adsorption kinetics results for D_2_O as compared to the case of H_2_O. Based on the fact that the rate of deceleration of the adsorption kinetics is different for the different proteins studied, the authors excluded the influence of viscosity effects (D_2_O has about 20% higher viscosity than H_2_O) and concluded that the observed adsorption behavior originates from a slower unfolding of the protein globules upon adsorption at the solid surface. Such a viscosity difference for H_2_O and D_2_O should not strongly affect the diffusion coefficient of a given protein, and indeed, as mentioned above, a comparison of independent investigations reveals only very small variations in the diffusion coefficient of BLG in H_2_O and in D_2_O,^49−51^ which is in line with predictions by molecular dynamics simulations showing that the effect of H_2_O–D_2_O exchange on the radius of gyration of BLG is less than 1%.^52^ Furthermore, the protein diffusivity in the bulk may be affected by changes in the size of the aqueous protein (molecular structure) and the net charge due to changes in pH. Note that under the current solvent conditions and protein concentration, BLG is expected to exist predominantly as a dimer in aqueous solutions, but there is evidence that BLG dimers dissociate into monomers upon adsorption at the W/A interface.^25^ For BLG in D_2_O, it was found that the increase in pH (without pD correction) in a wide range (from pH 3 to pH 11) leads to a nearly 2-fold decrease in the diffusion coefficient (from ∼13.3 × 10^–11^ to ∼6.5 × 10^–11^ m^2^/s).^51^ However, the results in Figure 4 suggest that neither isotopic effects nor the ensuing differences in the acid/base equilibrium (pH 7.0 in H_2_O and pD ≈ 7.4 in D_2_O) influence the adsorption behavior of BLG in region I. Apparently, in this initial stage of adsorption, BLG molecules adsorb at the interface in a diffusion-controlled regime in a manner that is insensitive to any possible differences in their molecular properties in the bulk due to the isotopic and pH effects considered.

In region II, the isotopic effect on the adsorption and dilational rheological behaviors of the studied BLG layers becomes visible, which implies a change in the adsorption mechanisms from diffusion-controlled to a presumably mixed diffusion-kinetic regime. Then it seems that the above considered hypothesis of Grunwald et al.^14^ can be reasonably accepted in the case of protein adsorption at the W/A interface as well. Here we hypothesize that the degree of conformational changes of a protein globule upon adsorption is related to the globule’s stability in the bulk. Then a protein globule with a higher stability in the bulk is supposed to undergo conformational changes that are weaker upon adsorption than a protein globule with a more flexible structure in the bulk. Indeed, it was shown for BLG and BSA that a distortion of the globular tertiary structure attained in the bulk (at constant pH) due to the action of denaturing agents (e.g., urea) enhances the adsorption dynamics but at the same time inhibits the dilational elasticity of the interfacial layers.^53,54^ Hence, such an analogy could serve as an explanation for the concomitant deceleration of the adsorption kinetics on one hand (Figure 4) and the enhancement of the elastic rheological behavior (Figure 5) on the other hand, as observed for the case of D_2_O in comparison to H_2_O (and ACMW). Along these lines, the analogy to the surface behavior of BLG at different pH values due to pH-induced changes in the globular stability is not straightforwardly relevant because such changes are accompanied by concomitant variations of the protein net charge, making the problem more complex. And indeed, it was shown that the surface EoS for BLG at the W/A interface is pH-dependent.^25^ It should be noted here that the E(Π(t))_g,f_ results in Figure 5 suggest slight changes in the EoS between the cases of D_2_O and H_2_O (and ACMW) under the assumption of a constant protein net charge. A surprising result is the crossover of the D_2_O curves with those for H_2_O (and ACMW), which occurs at the end of region II. At the current stage of investigation, we cannot provide an unambiguous explanation of the observed action of the solvent isotopic effect on the rheological behavior of BLG in region II; this may be achieved through additional experimental and theoretical work on the problem. Moreover, at the moment, we do not have direct information on how the solvent isotopic effect influences the dimeric state of BLG and how this might be related to our findings. It should be noted that buoyancy effects due to isotopic substitution in the aqueous medium are generally negligible for protein solutions on the experimentally relevant length scales as briefly outlined in the Supporting Information and therefore cannot be responsible for the discussed observations.

The discussed scenario taking place in region II does not hold for region III. The establishment of the surface pressure at very similar values for all isotopic contrasts within this final region suggests that the action of the isotopic effect relaxes at sufficiently long times of adsorption, namely, after about 10 h. Note that, according to theoretical predictions,^25^ in region III, the BLG surface monolayer is in the vicinity of its state of saturation. This means that the detected slight decrease in the average value of the dilational viscoelasticity modulus E within the linear viscoelasticity plateau region in the E(g)_f,Π_ dependences for the case of D_2_O (Figure 6b) should originate from weak aqueous isotope-induced modulation of the in-plane interactions among adsorbed protein molecules within the tightly packed two-dimensional protein network. On the other hand, such in-plane interactions among adsorbed protein molecules seem to be virtually insensitive to the solvent isotopic effect at larger amplitudes within the nonlinear viscoelasticity regime. This may give only qualitative evidence about the extent of the action of the solvent isotopic effect on the protein in-plane interactions, which are presumably determined by the hydrophobic effect and the strength of the hydrogen bonds. Whatever the detailed physicochemical background of this phenomenon, its action is comparatively weak, and it virtually does not affect the shape of the frequency dependences E(f)_g,Π_ (Figure 6a), which suggests no changes in the mechanism of stress response to area expansion/compression strains. Moreover, any differences in the stress response for the different isotopic contrasts virtually disappear within the nonlinear viscoelasticity regime. Therefore, one may conclude that the effect of isotopic substitution in the aqueous medium on the adsorption and dilational rheological behaviors of BLG is negligible within region III.

## 5 Conclusions

The analysis of the experimental data on the adsorption and surface dilation rheological properties of BLG layers at W/A interfaces allows for the conclusion that one can reasonably safely perform complementary adsorption experiments with BLG using H_2_O, D_2_O, and practically any H_2_O/D_2_O mixture with respect to a required time window of adsorption that ensures an almost complete absence of the observed isotopic effect; for the extreme case of pure D_2_O and under the studied solution composition, this time window is of about 10 h. However, one should keep in mind that changes in pH as well as in the concentrations of protein and electrolyte could affect such a temporal limit. This fact is actually not a problem because the proposed approach is based on a simple experiment (readily applicable also to water/oil interfaces), and thus it may conveniently serve as a useful tool for designing the time window of various experimental protocols involving adsorbing proteins from any H_2_O/D_2_O mixtures in parallel to conventional experiments in H_2_O. If the finding that the exchange of H_2_O by ACMW practically does not affect the interfacial behavior of BLG is proven for other proteins, then it may appear as a general rule, which validates the adequacy of using ACMW in protein adsorption studies. The use of ACMW in neutron reflectometry is crucial for resolving the surface excess Γ of adsorbed molecules at W/A interfaces with high accuracy.^1,2^ This is especially useful for the so-called low-Q_z_ analysis method,^55^ which is used to follow the adsorption kinetics Γ(t) on minute time scales.^2,56,57^ On the other hand, resolving the subnanometer layer thickness of adsorbed molecules requires the use of D_2_O.^1,2,38^ Hence, in neutron reflectometry studies, the proposed experiment would be recommended as a routine test to determine the minimum time of adsorption required for the attenuation of eventual isotopic effects.

## Supporting Information Available

Details on the experimental protocols; supporting data; tabulated data for the different dilational moduli and the corresponding S factors (H_2_O, D_2_O, and ACMW); and a brief discussion of buoyancy effects (PDF)
