# Supplementary material for: Effects of Aqueous Isotopic Substitution on the Adsorption Dynamics and Dilational Rheology of β-Lactoglobulin Layers at the Water/Air Interface
Source: J Phys Chem B. 2024 Mar 12;128(11):2821–30. doi: 10.1021/acs.jpcb.3c08417 (PMC10961727; doi:10.1021/acs.jpcb.3c08417)
Supplement: Supplementary file 1 — jp3c08417_si_001.pdf [file jp3c08417_si_001.pdf]

Supporting information

Effects of Aqueous Isotopic Substitution on Adsorption Dynamics and Dilational Rheology of β-Lactoglobulin Layers at the Water/Air Interface

Georgi G. Gochev,[1,2,*] Emanuel Schneck,[3] Reinhard Miller[3]

[1] *Jerzy Haber Institute of Catalysis and Surface Chemistry, Polish Academy of Sciences, 30239 Krakow, Poland*

[2]*Institute of Physical Chemistry, Bulgarian Academy of Sciences, 1113 Sofia, Bulgaria*

[3] *TU Darmstadt, Institute for Condensed Matter Physics, 64289 Darmstadt, Germany*

[*] Corresponding author, e-mail: georgi.gochev@ikifp.edu.pl

Experimental

In dilational rheometry, harmonic perturbations of the available area are be applied at equilibrium or quasi-equilibrium states of the adsorbed layer on the drop/bubble surface. Figure S1 shows a representative experiment of the oscillating area A(t) of a bubble in solution and the corresponding surface tension response γ(t). The latter can be easiest explained by the complex quantity $E(i\omega)$:

$$E(i\omega) = E'(\omega) + iE''(\omega) = \frac{F\{\Delta\gamma(t)\}}{F\{\Delta\ln A(t)\}}$$

where F stands for the Fourier transform and ω [rad.s] = 2πf [Hz] is the angular frequency. The two parameters E' and E'' are the real and imaginary part of the complex viscoelasticity modulus E and represent the elastic and viscous contributions, respectively.

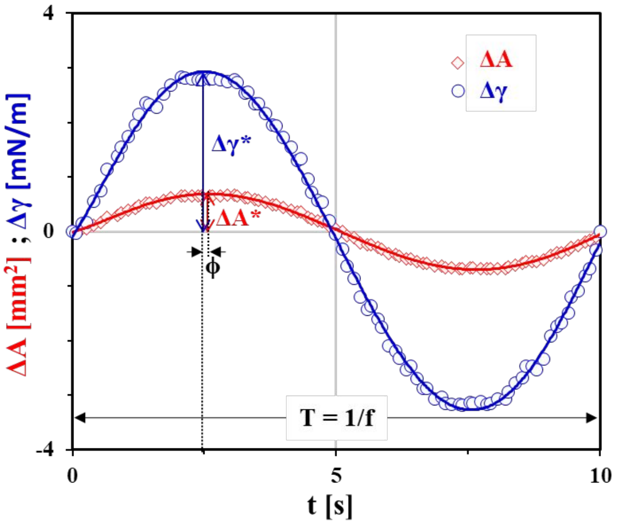

Fig. S1. A full period of a sinusoidal bubble oscillation presented in terms of the normalized bubble area $\Delta A = A(t) - A_0$ and the corresponding surface tension response $\Delta\gamma = \gamma(t) - \gamma_{A0}$, γ_{A0} is the quasi-static surface tension at the undisturbed area A_0. Legend: T - period of oscillation (here 10 s), f - frequency (here 0.1 Hz), ϕ - viscous phase angle, ΔA* - amplitude of area oscillation, Δγ* - amplitude of the surface tension response; lines are guide to the eye. The degree of area deformation (amplitude) g is defined as: $g \equiv \Delta A/A_0$ [×100, %].

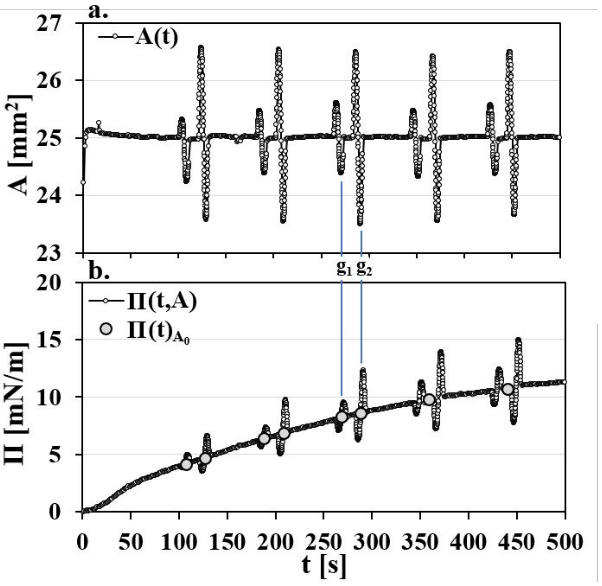

Fig. S2. A representative experiment with a buoyant bubble in a H_2O solution of BLG at $C_{BLG} = 1$ μM, C_{buff} = 10 mM and pH 7. **a)** Surface area A(t); and **b)** dynamic surface pressure Π(t) for the initial interval of adsorption; the larger filled symbols in (b) are selected Π-t datapoints at an undisturbed surface area of A_0 = 25 mm^2. Bubble area oscillations were performed at f = 0.1 Hz, g_1 = 2.5 %, g_2 = 6.5 %.

Fig. S3-a shows a series of single oscillations grouped in three types of sets: 1) a set of oscillations at different frequencies, called 'frequency sweep' (f-sweep) at fixed $g_1 \approx 2.5$ %; 2) a set of oscillations at different deformation amplitudes, called 'amplitude sweep' (g-sweep) at fixed f = 0.1 Hz; and 3) a second f-sweep at fixed $g_2 \approx 6.5$ %. Note that this oscillation protocol was applied only at sufficiently long times of adsorption, when the surface tension (surface pressure) after the oscillation cycle remains virtually the same as that at the beginning of the cycle (see the Π-t plot in Fig. S3-a), and thus we assume that the obtained results correspond to quasi-static adsorption states with certain local steady state Π-values. At the longest adsorption times, when the surface pressure tends to level off at plateau values (close to equilibrium), f-sweeps (Fig. S3-b) and g-sweeps (Fig. S3-c) were applied in wider frequency and amplitude ranges, respectively.

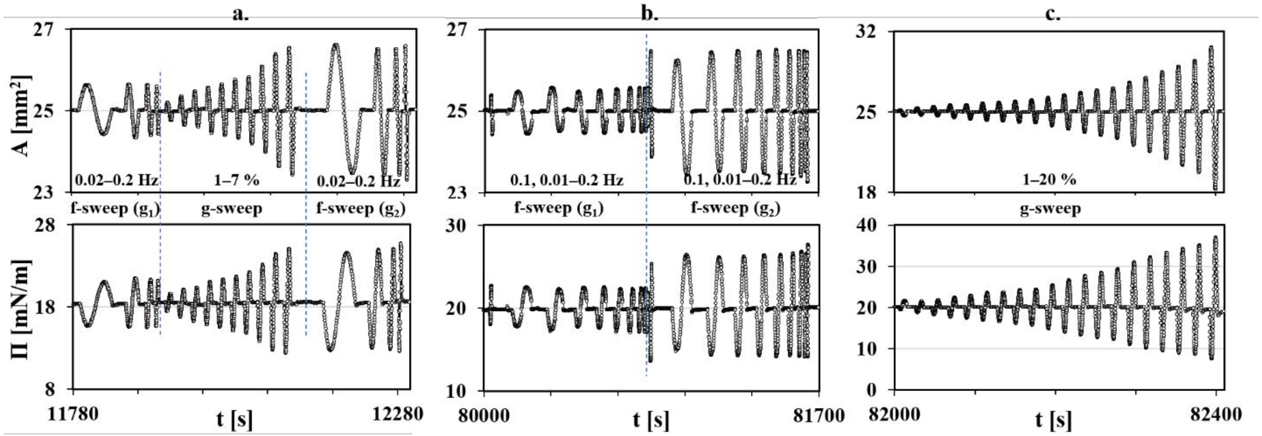

Fig. S3. The same experiment as in Fig. S2. **a)** A set of two frequency sweeps (f-sweep) in the range 0.02–0.2 Hz at two area deformation amplitudes of $g_1 \approx 2.5$ % and $g_2 \approx 6.5$ %, and an amplitude sweep (g-sweep) in the range 1–7 % at a frequency of f = 0.1 Hz. **b)** f-sweeps in a wider range (0.01–0.2 Hz). **c.** g-sweeps in a wider range (1–20 %).

Results

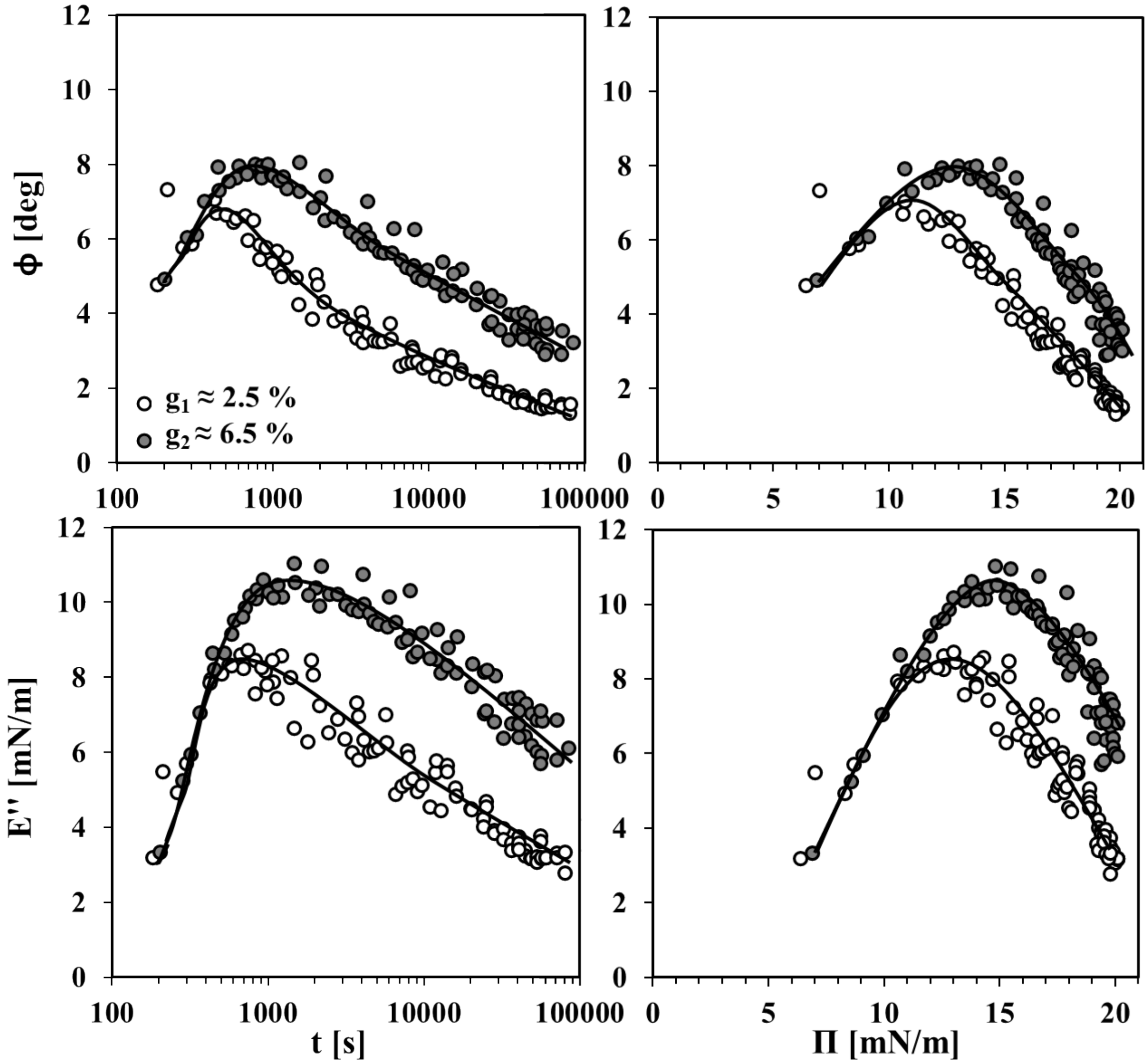

Fig. S4. Extension of Fig. 1 from the main text; the same experiment as in Figs. S2,S3. Dependencies of the phase angle $\phi(t)_{g,f}$ and $\phi(\Pi(t))_{g,f}$, and the imaginary part of the complex dilational viscoelasticity modulus $E''(t)_{g,f}$ and $E''(\Pi(t))_{g,f}$; $f = 0.1$ Hz, $g_1 \approx 2.5$ % and $g_2 \approx 6.5$ %. Lines are guides to the eye.

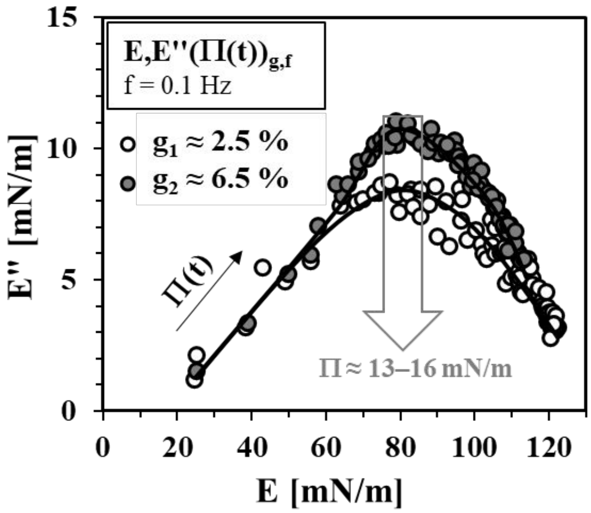

Fig. S5. E''(E(Π(t))$_{g,f}$ plots; lines are guides to the eye.

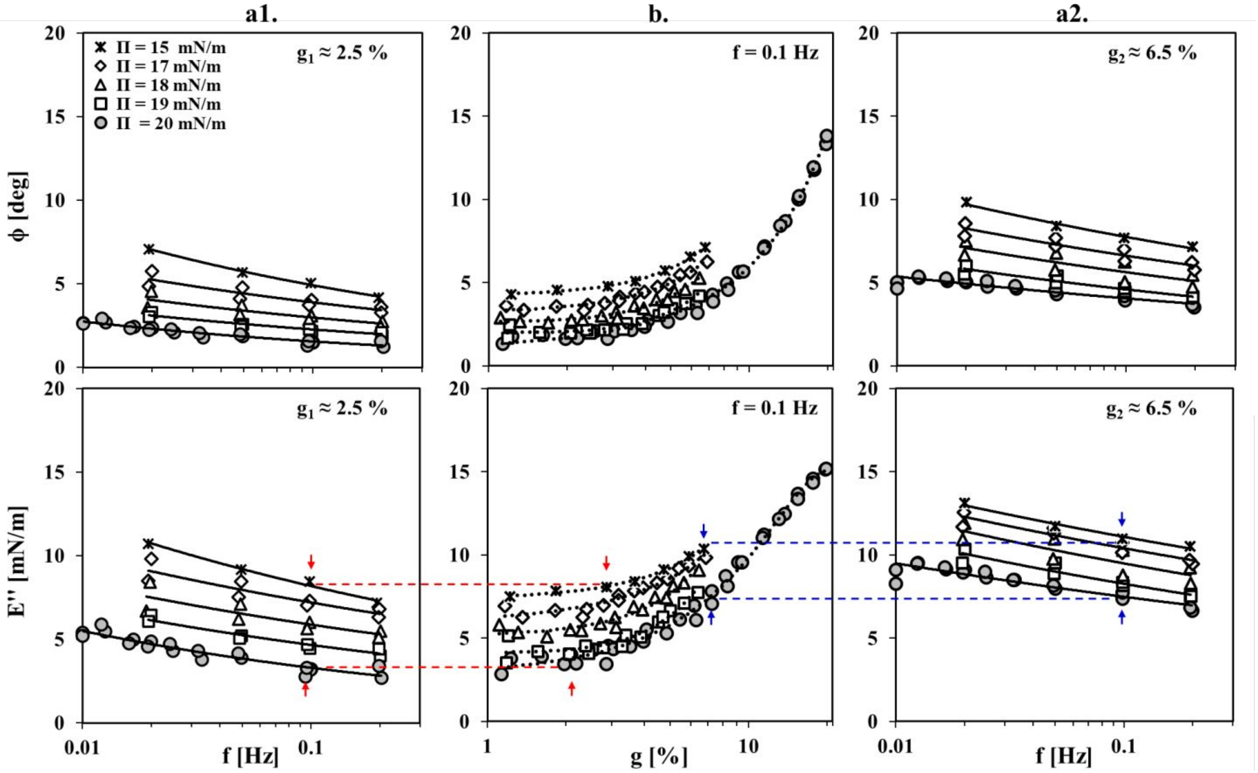

Fig. S6. Extension of Fig. 2 from the main text; the same experiment as in Figs. S2-S5. **a1,a2)** Frequency dependencies $\phi(f)_{g,\Pi}$ and $E''(f)_{g,\Pi}$ at different surface pressures Π and amplitudes g_1 an g_2; the data in the narrower f-range correspond to the f-sweeps in Fig. S3-a measured in the course of adsorption and the data in the wider f-range correspond to the f-sweeps in Fig. S3-b measured at long adsorption times. **b)** Area deformation amplitude dependencies $\phi(g)_{f,\Pi}$ and $E''(g)_{f,\Pi}$ at different surface pressures Π; the data in the narrower g-range correspond to the g-sweeps in S3-a measured in the course of adsorption and the data in the wider g-range up to 20 % correspond to the g-sweeps in S3-c measured at long adsorption times; f = 0.1 Hz. (H_2O solution of BLG at C_{BLG} = 1 μM, C_{buff} = 10 mM and pH 7).

Each of the moduli E_{LE} or E_{LC} is determined by the slope of the linear relation between the origin (0.0) and the coordinates (Δγ,g) at the respective loop apex (well-evident in Fig. S7-d). Each of the moduli E_{ME} and E_{MC} is determined by the local tangent at g = 0 to the experimental data. The numerical analysis was made in Microsoft Excel with an accuracy of ±1 mN/m.

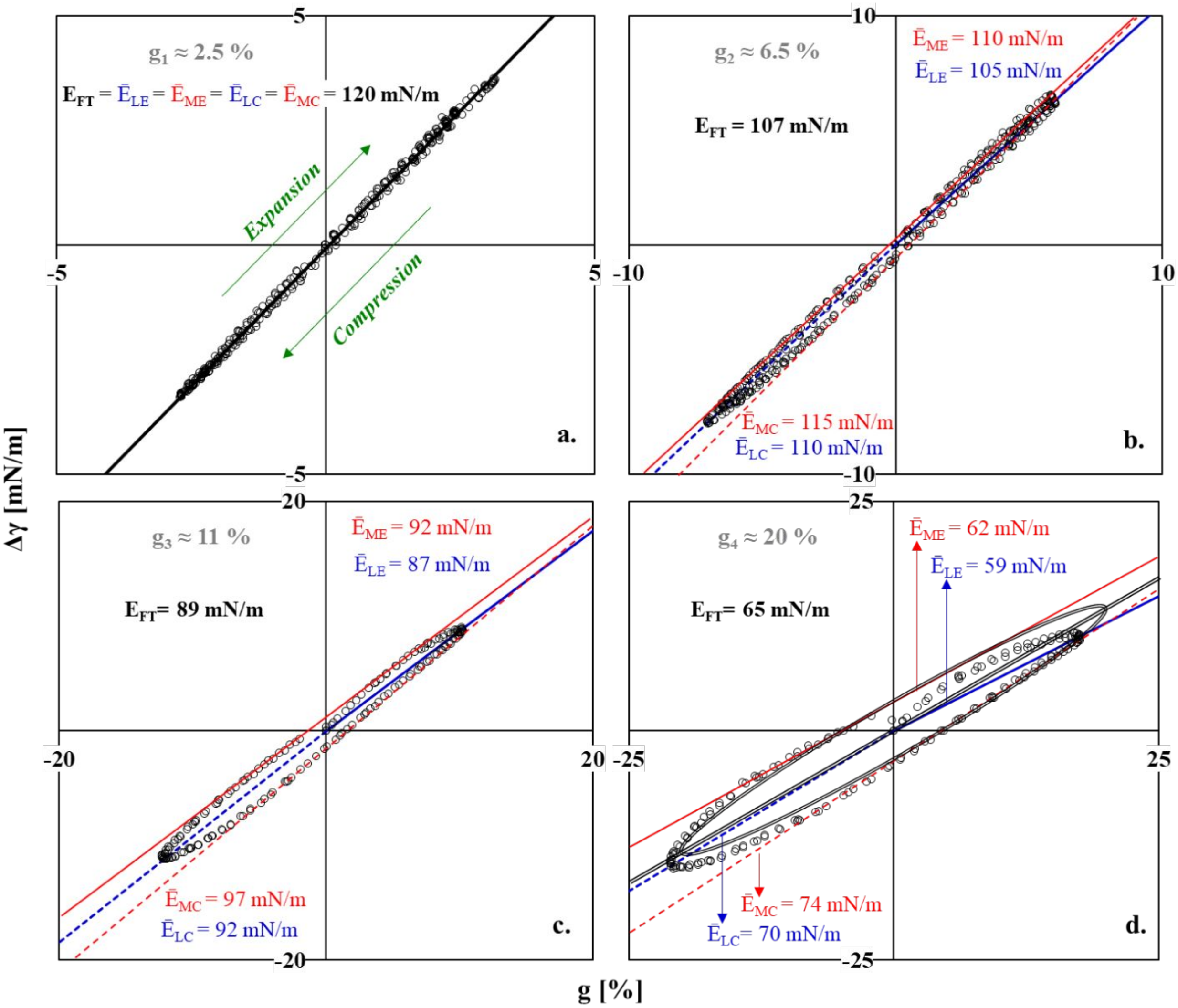

Fig. S7. Results at a steady state (Π ≈ 20 mN/m) from the two long (20–22 hours) experiments in Fig. 1. Lissajous plots Δγ-g constructed from the raw data γ(A(t)) measured during bubble oscillations in large-range g-sweeps; in (a,b) data from large-range f-sweeps are also included. Hence, each graph in (a,b) contains 4 single plots and each graph in (c,d) contains 2 single plots. Lines: (□) $\bar{E}_{LE}$, (---) $\bar{E}_{LC}$, (□) $\bar{E}_{ME}$, (---) $\bar{E}_{MC}$; the straight double-line through the origin (0,0) is the long axes of an ellipse, which is the first-harmonics of the Fourier transform fits to the raw data.

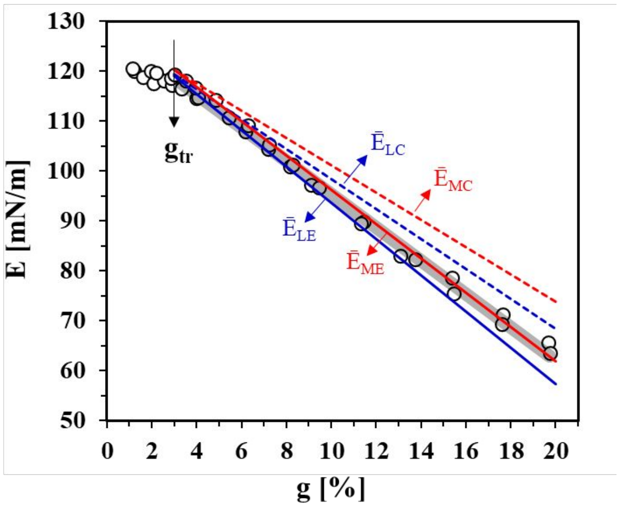

Fig. S8. Comparison of the experimental $E(g)_{f,\Pi}$ dependences for the different E moduli. Symbols are the E_{FT} moduli measured with the bubble profile analysis tensiometer; the grey-shaded ribbon through the symbols follows a linear regression of the data at $g > g_{tr}$. The lines (□) $\bar{E}_{LE}$, (---) $\bar{E}_{LC}$, (□) $\bar{E}_{ME}$, (---) $\bar{E}_{MC}$ are linear regressions (details are given in the text).

Table S1. Calculated values for the different dilational moduli and the S-factors.

g [%]	E_{FT} [mN/m]	$\bar{E}_{ME}$ [mN/m]	$\bar{E}_{LE}$ [mN/m]	S_E [-]	$\bar{E}_{MC}$ [mN/m]	$\bar{E}_{LC}$ [mN/m]	S_C [-]
				**H_2O**			
**2.5**	$E_{FT} = \bar{E} = 120$; S (-)						
**6.5**	110	110	105	– 0.05	113	109	– 0.04
**11**	89	92	87	– 0.06	97	92	– 0.05
**20**	65	62	59	– 0.05	74	70	– 0.06
				**D_2O**			
**2.5**	$E_{FT} = \bar{E} = 112$; S (-)						
**6.5**	104	108	102	– 0.06	110	104	– 0.06
**20**	67	62	62	0*	76	70	– 0.09*

* Of notable comparative difference

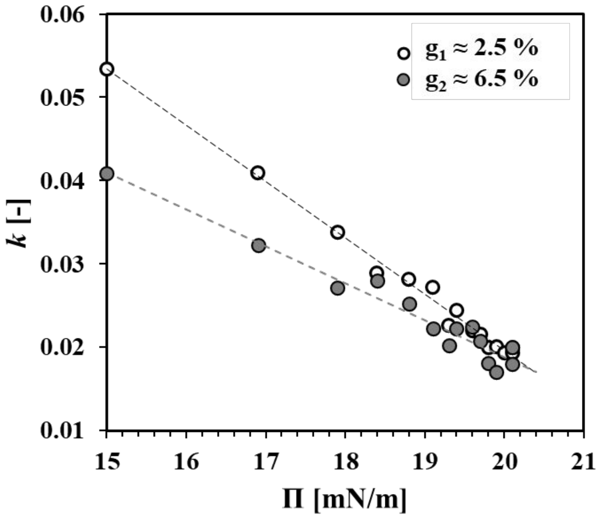

Fig. S9. Surface pressure dependence of the exponent *k* in the power law E ~ f^k fittings in Fig. 2.

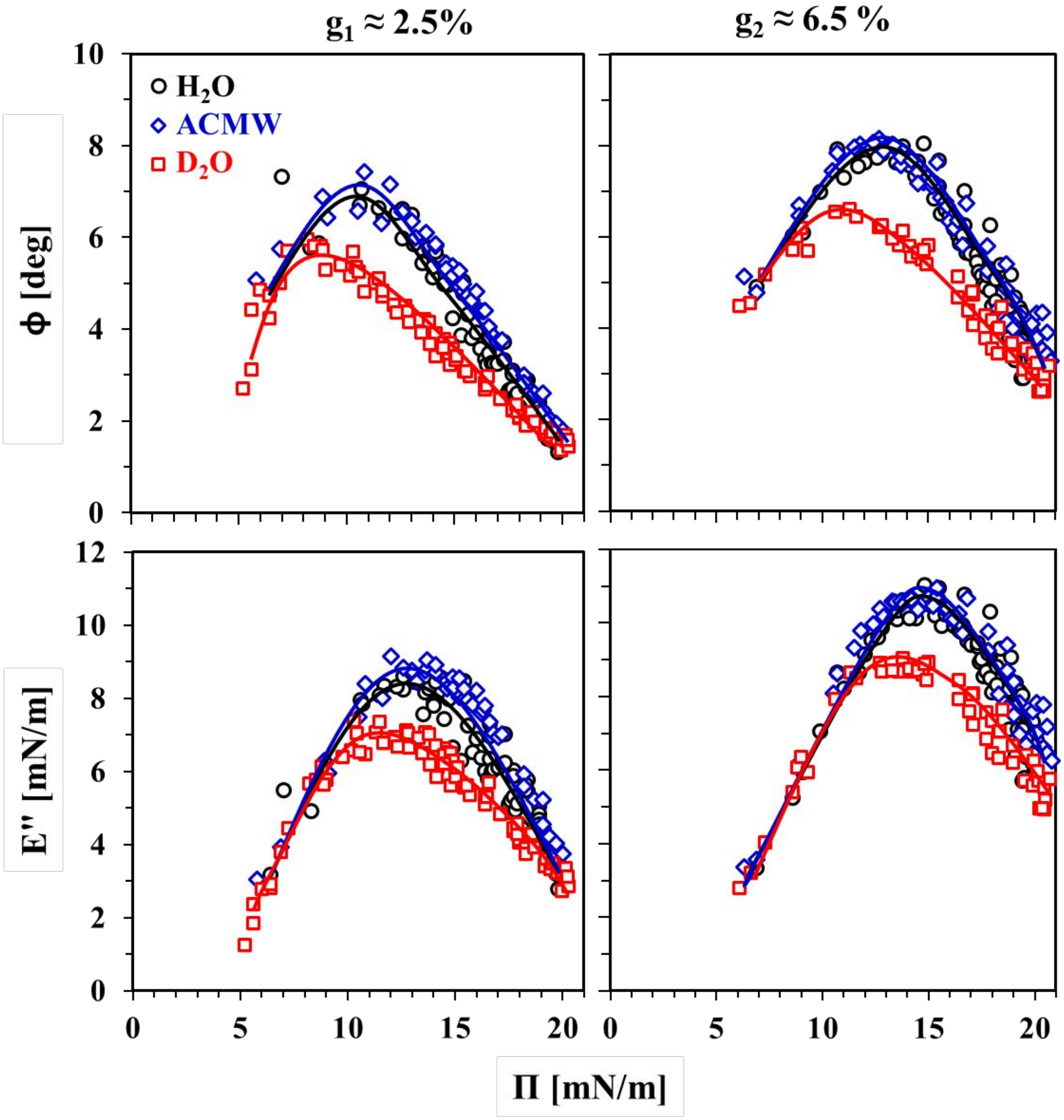

Fig. S10. Extension of Fig. 5 from the main text; lines are guides to the eye.

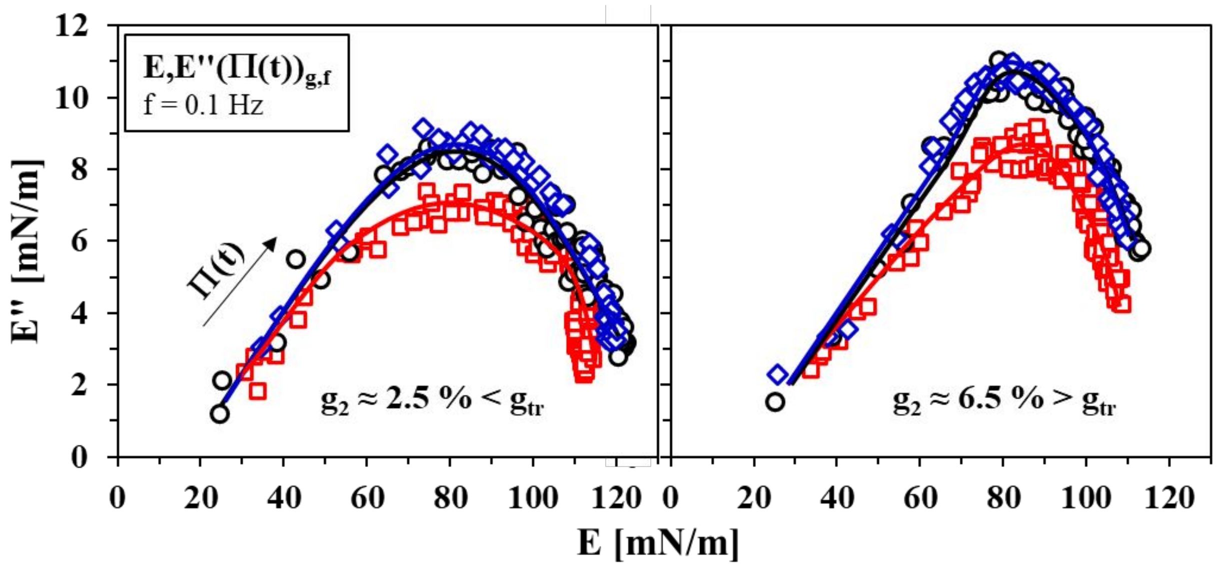

Fig. S11. $E''(E(\Pi(t))_{g,f}$ plots; lines are guides to the eye.

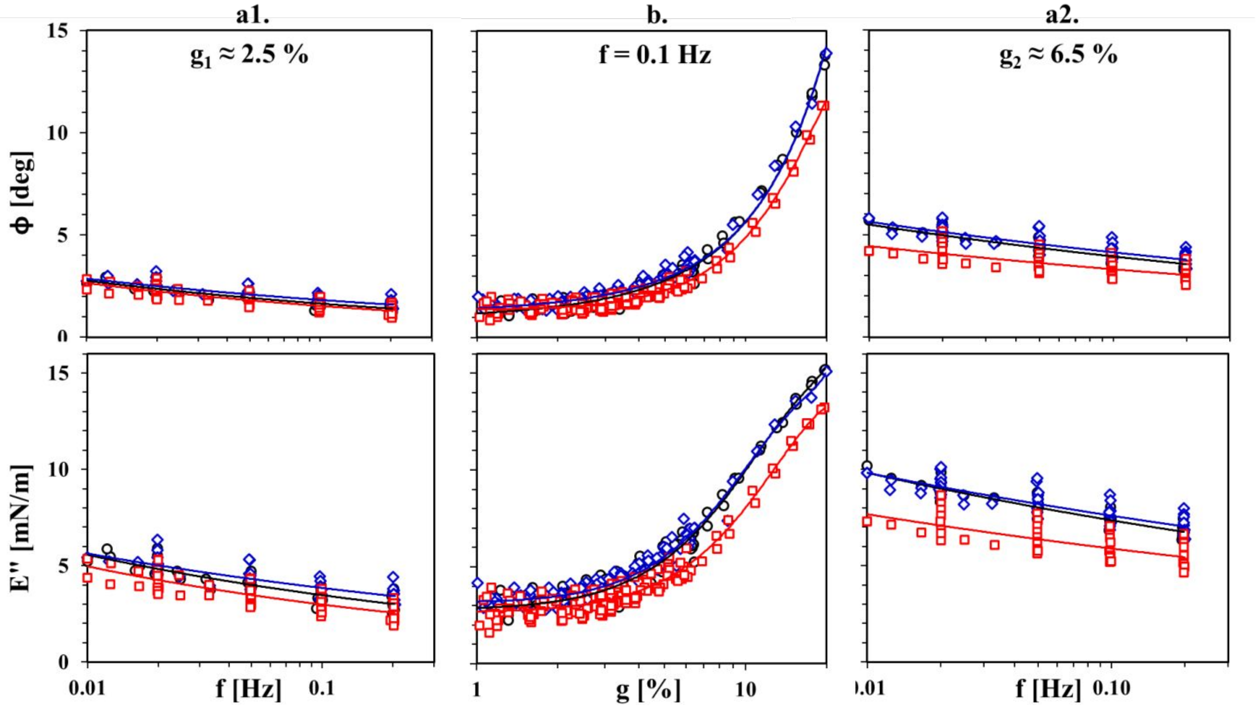

Fig. S12. Extension of Fig. 6 from the main text; lines are guides to the eye.

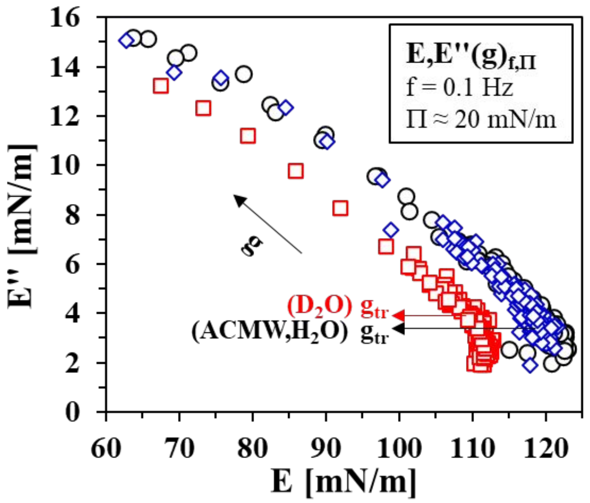

Fig. S13. E"(E(g))$_{f,\Pi}$ at a steady state; lines are guides to the eye.

Discussion

Following from the Boltzmann distribution, $p(h) = e^{-m_{\mathrm{eff}}gh/(k_{\mathrm{B}}T)} = e^{-h/h_0}$, the vertical length scale for protein concentration variations due to gravity is $h_0 = (k_{\mathrm{B}}T)/(m_{\mathrm{eff}}g)$, where k_B is the Boltzmann constant, g is the fall acceleration, T is the temperature, and $m_{\mathrm{eff}} = V_{\mathrm{P}}(\rho_{\mathrm{P}} - \rho_{\mathrm{W}})$ is the buoyancy-corrected protein mass, where V_{P} is the protein volume (~100 nm^3), ρ_{P} is the mass density of protein (for BLG, $\rho_{\mathrm{P}} \approx 1.34$ g/cm^3 in H_2O and $\rho_{\mathrm{P}} \approx 1.36$ g/cm^3 in D_2O), and ρ_{W} is the mass density of water (≈1.0 g/cm^3 for H_2O and ≈1.1 g/cm^3 for D_2O). Irrespective of the H_2O/D_2O mixture, h_0 is on the order of 10 m, meaning that, on the experimental length scales (<< 1 m), the influence of buoyancy on concentration variations is negligible.
